# Neuroforamen stenosis remains a challenge in conventional computed tomography and new dual-energy techniques

**DOI:** 10.1038/s41598-022-10673-3

**Published:** 2022-04-23

**Authors:** Ann-Kathrin Ditges, Torsten Diekhoff, Nils Engelhard, Maximilian Muellner, Matthias Pumberger, Friederike Schömig

**Affiliations:** 1 Department of Radiology, Charité – University Medicine Berlin, Charitéplatz 1, 10117 Berlin, Germany; 2 Center for Musculoskeletal Surgery, Charité – University Medicine Berlin, Charitéplatz 1, 10117 Berlin, Germany

**Keywords:** Medical research, Computed tomography, Magnetic resonance imaging

## Abstract

Lumbar foraminal stenosis may be caused by osseous and soft tissue structures. Thus, both computed tomography (CT) and magnetic resonance imaging (MRI) play a role in the diagnostic algorithm. Recently, dual-energy CT (DECT) has been introduced for the detection of spinal disorders. Our study’s aim was to investigate the diagnostic accuracy of collagen-sensitive maps derived from DECT in detecting lumbar foraminal stenosis compared with standard CT and MRI. We retrospectively reviewed CT, DECT, and MRI datasets in patients with vertebral fractures between January 2015 and February 2017. Images were scored for presence and type of lumbar neuroforaminal stenosis. Contingency tables were calculated to determine diagnostic accuracy and interrater agreement was evaluated. 612 neuroforamina in 51 patients were included. Intraclass correlation coefficients for interrater reliability in detecting foraminal stenoses were 0.778 (95%-CI 0.643–0.851) for DECT, 0.769 (95%-CI 0.650–0.839) for CT, and 0.820 (95%-CI 0.673–0.888) for MRI. Both DECT and conventional CT showed good diagnostic accuracy in detecting lumbar foraminal stenosis but low sensitivities in detecting discoid stenosis. Thus, even though previous studies suggest that DECT has high diagnostic accuracy in assessing lumbar disc pathologies, we show that DECT does not provide additional information for detecting discoid stenosis compared with conventional CT.

## Introduction

With a reported prevalence of 8–26%, lumbar foraminal stenosis is a common cause of lumbar radiculopathy^1–3^. Underlying pathologies include disc degeneration with loss of disc height, facet or ligamentous hypertrophy, and osteophytes^4^. Foraminal stenosis causes irritation of a specific nerve root, which in turn leads to symptoms such as pain or sensory loss in the leg or possible motor function loss depending on the spinal levels involved^5^. Thus, foraminal stenosis is an important differential diagnosis in patients with radiculopathy. In addition to a thorough physical examination, patients undergo magnetic resonance imaging (MRI) to evaluate the intervertebral foramen as the site of nerve root irritation. However, published reports on imaging-based grading and classification of lumbar foraminal stenosis are rare. In 2010, Lee et al. proposed an MRI-based grading system including the type of stenosis, amount of fat obliteration, and presence of nerve root compression (Table 1)^6^. While computed tomography (CT) may be more accurate in diagnosing foraminal stenoses caused by osseous structures such as osteophytes, its value in detecting foraminal stenoses caused by the intervertebral disc remains limited.

**Table 1:** Classification of lumbar foraminal stenosis as proposed by Lee et al.^6^.

Grade	Characteristics
0: no foraminal stenosis	No perineural fat obliteration
1: mild foraminal stenosis	Perineural fat obliteration in vertical or transverse direction No evidence of morphologic change in nerve root
2: moderate foraminal stenosis	Perineural fat obliteration in all four directions No evidence of morphologic change in nerve root
3: severe foraminal stenosis	Nerve root collapse or morphologic change

Recently, dual-energy computed tomography (DECT) has been introduced for the detection of spinal disorders. While initially, DECT was predominantly used in the imaging of gout, by using virtual noncalcium images it has been shown to be able to visualize bone marrow edema, for example in patients with vertebral fractures^7–9^. As another application of DECT imaging, collagenous structures may be depicted by a so-called three-material-decomposition algorithm^10^. This collagen-sensitive mapping has been shown to be feasible in the imaging of ligaments and tendons^11–13^ and, more recently, of the intervertebral disc allowing a visualization of spinal pathologies such as intervertebral disc herniations^14^.

As lumbar foraminal stenosis may be caused by both osseous and soft tissue structures, visualization of the disc by CT might prove to be especially helpful in patients with contraindications to MRI or who need a CT scan for other indications. Even though previous studies have shown high sensitivity and specificity of collagen-sensitive maps based on DECT in the detection of lumbar disc pathologies^14–16^, to our knowledge there are no studies evaluating their benefit to standard CT reconstructions in assessing both discoid and osseous lumbar foraminal stenoses.

Thus, the aim of this retrospective study was to investigate the diagnostic accuracy of collagen-sensitive mapping using DECT in the detection of lumbar foraminal stenosis compared with standard CT alone and MRI in a feasibility approach.

## Methods

### Patients and ethics approval

The study was approved by the ethics committee of Charité – Universitätsmedizin Berlin (EA1/372/14). The need of informed consent was waived by the ethics committee of Charité – Universitätsmedizin Berlin. We retrospectively reviewed patients who had been prospectively enrolled between January 2015 and February 2017 with acute back pain and vertebral fractures visible on radiographs and had undergone both DECT and MRI of the lumbar region of the spine.

### Image acquisition

DECT of the lumbar spine was performed on a 320-row single-source CT scanner (Canon Aquilion ONE Vision Edition; Canon Medical Systems, Tochigi, Japan) and included both a scanogram and DECT with sequential volume acquisition of two energy datasets (135 and 80 kVp). Rotation time was 0.275 s with a change-over time of 0.5 s between acquisitions. Exposure control was set to a standard deviation of 12. The wide volume mode was used if necessary.

MRI was performed on a clinical 1.5-T standard imager (MAGNETOM Avanto; Siemens Helthineers or MAGNETOM Symphony Vision; Siemens Healthineers) and included a T1-weighted (repetition time, 551 ms; echo time, 12 ms; acquisition time, 5 min 12 s) sequence and a short tau inversion recovery (STIR) sequence (repetition time, 6150 ms; echo time, 31 ms; inversion time, 150 ms; acquisition time, 4 min 15 s) with a slice thickness of 3 mm.

### Postprocessing

Primary DECT raw data were reconstructed to 135 kVp standard CT images with a slice thickness and interval of 0.5 mm using iterative reconstruction (AIDR-3D standard) and with a medium soft tissue kernel without beam hardening compensation (FC13) and a sharp bone kernel. Collagen maps were generated on the CT console (Dual Energy Image View, Version 6; Canon Medical Systems) using a collagen-specific gradient of 1.10. Secondary multiplanar reconstructions were computed from every CT volume dataset as 3-mm image stacks without overlap as primary series for image interpretation.

### Image reading

**Figure 1:**
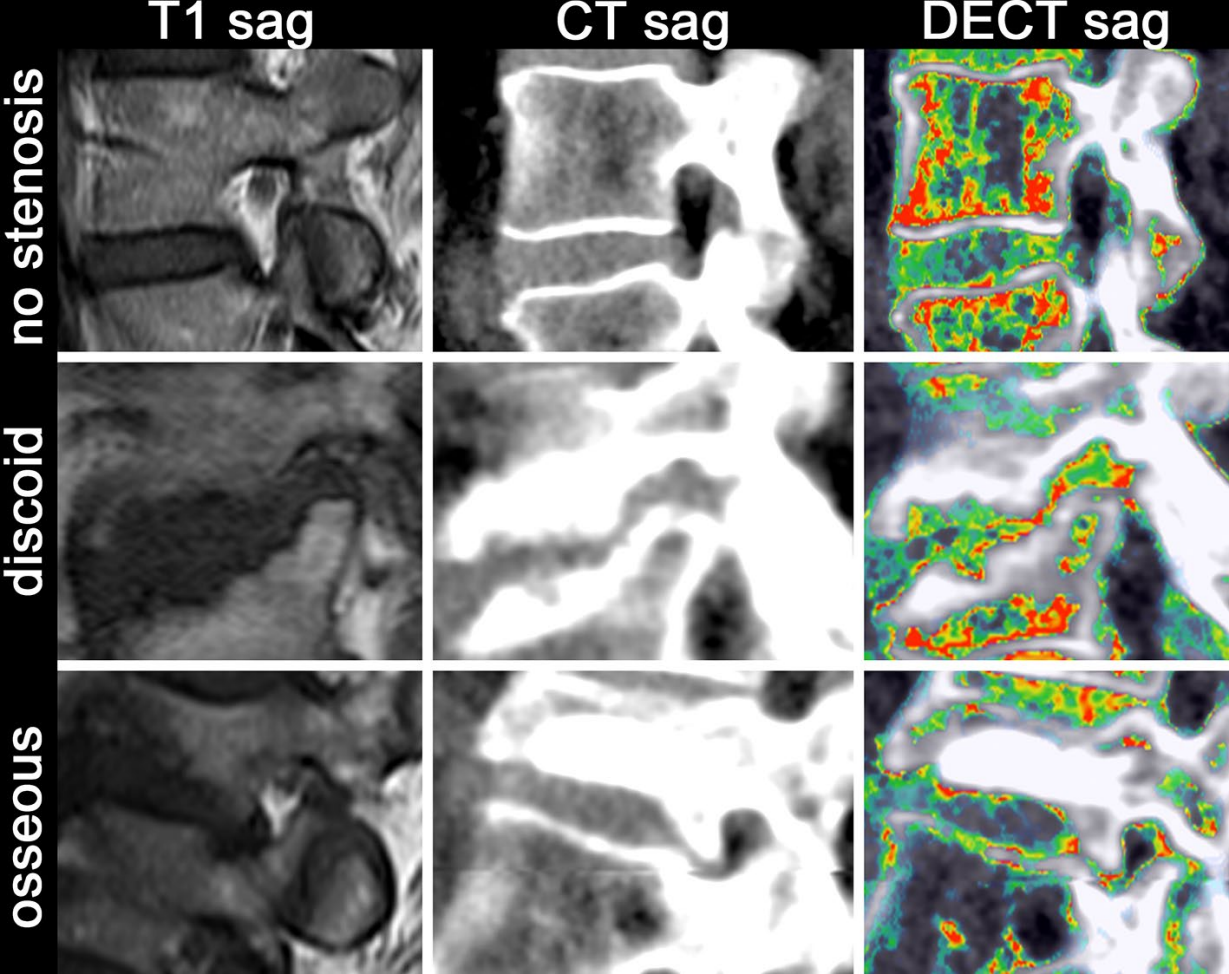
Imaging examples of no foraminal stenosis as well as discoid and osseous stenosis in (left to right) MRI, standard CT, and collagen-sensitive dual-energy reconstructions.

MRI, CT (reconstructed from DECT acquisition), and DECT images were separately anonymized and independently analyzed by two readers (reader 1, an orthopedic surgery resident with two years of experience; reader 2, a medical research student with one year of experience who was trained by a radiologist specializing in musculoskeletal imaging) on a workstation with a high-resolution monitor using Horos (version 3.3.5). Furthermore, a consensus scoring was performed by a radiologist specializing in musculoskeletal disorders with eleven years of experience and a spine surgeon with eleven years of experience. Readers had access to one modality at a time and were blinded to identifying and clinical information as well as findings of the other modalities. When scoring collagen-sensitive maps derived from DECT, readers had access to standard CT reconstructions. Scoring was performed in sagittal and oblique axial planes of all three modalities. Neuroforaminal stenoses were scored using a 4-point semiquantitative grading system based on the grading system proposed by Lee et al.^6^: 0, normal neuroforamen; 1, mild foraminal stenosis; 2, moderate foraminal stenosis; 3, severe foraminal stenosis (Table 1). Additionally, if a foraminal stenosis was detected, its type was scored (“osseous”, “discoid”, or “osseous and discoid”). Discoid stenosis was defined as a stenosis caused by discoid protrusion whereas osseous stenosis was defined as a stenosis caused by osteophytic protrusion in the foraminal zone or facet arthropathy (Fig. 1). Mixed osseous and discoid stenoses were defined as a combination of both. Foramina were defined as “cannot be assessed” in case of incomplete depiction of the foramen or in case of limited assessability due to metal artefacts.

### Data postprocessing

For the calculation of diagnostic accuracy, the scale “neuroforaminal stenosis” was dichotomized as follows: 0, no neuroforaminal stenosis; 1, neuroforaminal stenosis. To discriminate between osseous and discoid foraminal stenosis in the determination of diagnostic accuracy, the scale “type of foraminal stenosis” was dichotomized as follows: for discoid stenoses: 0, no neuroforaminal stenosis or osseous stenosis; 1, discoid or both osseous and discoid stenosis; for osseous stenoses: 0, no neuroforaminal stenosis or discoid stenosis; 1, osseous or both osseous and discoid stenosis.

### Statistical analysis

For the determination of interrater reliability between the three readings (reader 1, reader 2, consensus reading) for each imaging technique (MRI, CT, and DECT), two-way random single-measure intraclass correlation coefficients (ICCs) and 95% confidence intervals (95%-CIs) were calculated for the degree of neuroforaminal stenosis. For determining the interrater reliability for the type of foraminal stenosis, Fleiss’ kappa was calculated.

For dichotomized variables of the presence of foraminal stenosis, contingency table analysis was performed with MRI and CT as standard of reference. For dichotomized variables of the type of stenosis, contingency table analysis was performed with MRI as standard of reference for discoid stenosis and CT for osseous stenosis. In case of mixed discoid and osseous stenosis, both MRI and CT were used as standard of reference. Additionally, Cohen’s kappa was calculated to determine agreement between each imaging technique.

The statistical significance level for all tests performed was p < 0.05. Statistical analysis was performed using Excel (Microsoft, Version 16.42) and SPSS (IBM, Version 27).

## Results

### Patients

**Figure 2:**
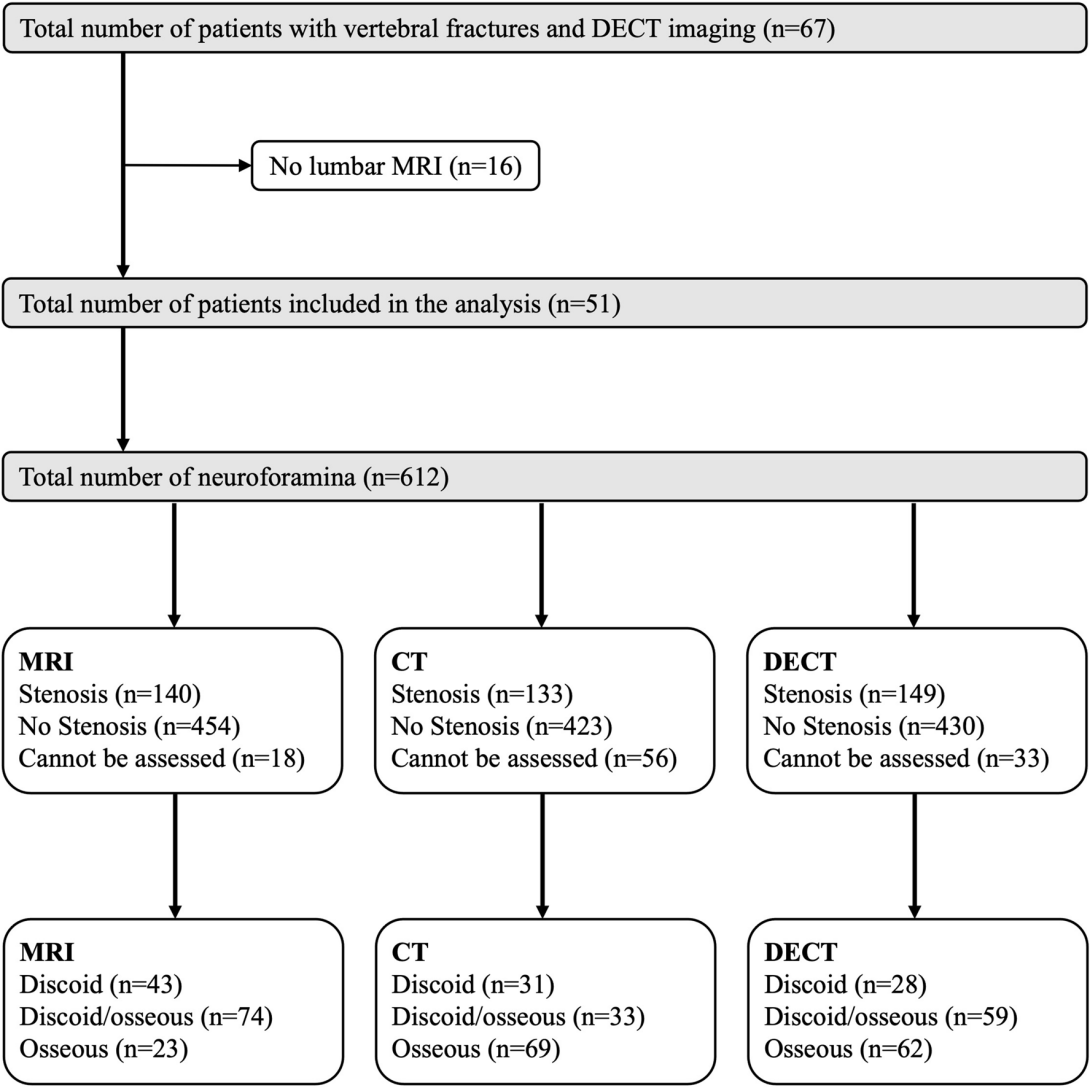
Patient flow chart. All patients who underwent DECT imaging were included while patients with missing lumbar MRI were excluded.

A total of 67 patients underwent DECT. Sixteen patients were excluded due to missing or incomplete lumbar MRI, yielding 51 included patients with a total of 612 lumbar neuroforamina. The number of included foraminal stenoses and the according type of stenosis per imaging modality are depicted in Fig. 2. The included patients had a mean age of 71 years ± 10. Mean time between CT/DECT and MRI was 23 days ± 79.

### Scoring

**Figure 3:**
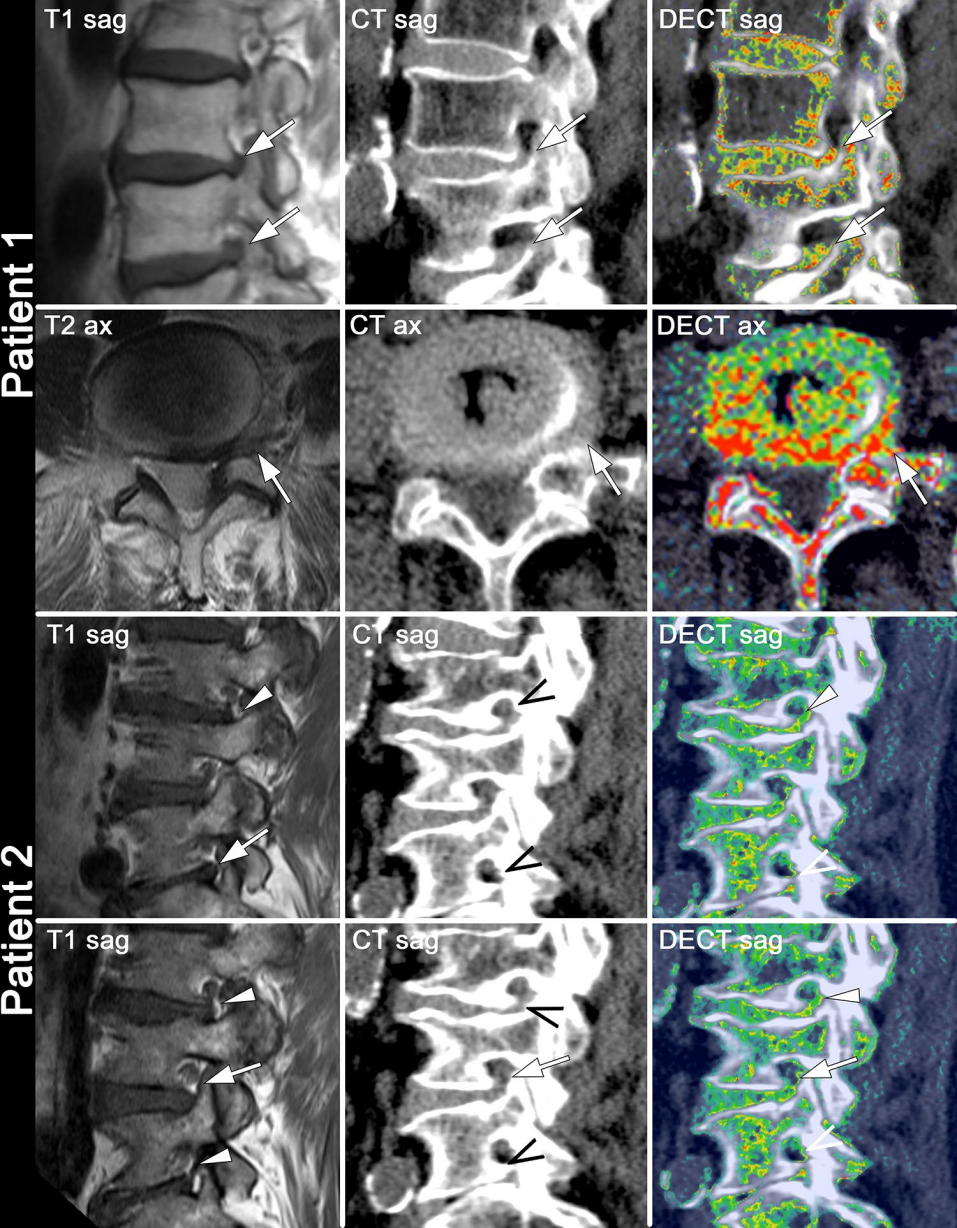
Imaging examples from two different patients. For each patient (left to right) MRI, standard CT, and collagen-sensitive dual-energy reconstructions are shown with the according consensus scoring. Arrows: discoid foraminal stenosis; arrowheads: mixed discoid/osseous foraminal stenosis; open arrowheads: osseous foraminal stenosis.

A representative example of a scored imaging dataset is shown in Fig. 3.

### Presence of lumbar foraminal stenosis

In the 612 neuroforamina scored, DECT was positive for lumbar foraminal stenosis in 149 cases, CT in 133 cases, and MRI in 140 cases. The number of neuroforamina scored as “cannot be assessed” was 34 for DECT, 52 for CT, and 18 for MRI. Results of the contingency table analysis with calculations of diagnostic accuracies for DECT and CT are compiled in Table 2. Cohen’s kappa was 0.635 (p < 0.001) for DECT, 0.795 (p < 0.001) for CT, and 0.770 (p < 0.001) for MRI.

**Table 2:** Contingency table analysis of the presence of foraminal stenosis for DECT, MRI, and CT with both MRI and standard CT as standard of reference.

	MRI/CT+	MRI/CT−	Total
DECT+	106	33	139
DECT−	44	351	395
Total	150	384	534
**SE**	**SP**	**PPV**	**NPV**
0.71	0.91	0.76	0.89

	MRI/CT+	MRI/CT−	Total
MRI+	123	13	136
MRI−	37	382	419
Total	160	395	555
**SE**	**SP**	**PPV**	**NPV**
0.77	0.97	0.90	0.91

	MRI/CT+	MRI/CT−	Total
CT+	117	16	133
CT−	26	377	403
Total	143	393	536
**SE**	**SP**	**PPV**	**NPV**
0.82	0.96	0.88	0.94

Data are given with 95% confidence intervals.

*SE* sensitivity, *SP* specificity, *PPV* positive predictive value, *NPV* negative predictive value, *MRI* magnetic resonance imaging, *CT* computed tomography, *DECT* dual-energy computed tomography, + discoid or mixed stenosis in MRI or osseous or mixed stenosis in standard CT, − discoid stenosis in standard CT, osseous stenosis in MRI, or no stenosis.

ICCs for interrater reliability were 0.778 (95%-CI 0.643–0.851) for DECT, 0.769 (95%-CI 0.650–0.839) for CT, and 0.820 (95%-CI 0.673–0.888) for MRI.

### Type of lumbar foraminal stenosis

In the detection of discoid foraminal stenoses, Cohen’s kappa for the agreement between MRI and DECT was 0.350 (p < 0.001) and 0.369 (p < 0.001) between MRI and CT. In the detection of osseous foraminal stenoses, Cohen’s kappa for the agreement between CT and DECT was 0.580 (p < 0.001) and 0.350 (p < 0.001) between CT and MRI. Contingency table analysis for the detection of discoid foraminal stenoses yielded a sensitivity of 0.42 and specificity of 0.91 for DECT and a sensitivity of 0.38 and specificity of 0.94 for CT (Table 3). Contingency table analysis for the detection of osseous foraminal stenoses yielded a sensitivity of 0.71 and specificity of 0.90 for DECT and a sensitivity of 0.47 and specificity of 0.92 for MRI (Table 4). In the detection of mixed foraminal stenoses, Cohen’s kappa for the agreement between DECT and MRI/CT was 0.336 (p < 0.001), 0.452 (p < 0.001) between CT and MRI/CT, and 0.625 (p < 0.001) between MRI and MRI/CT. Contingency table analysis for the detection of mixed stenoses yielded a sensitivity of 0.41 and specificity of 0.93 for DECT, a sensitivity of 0.39 and specificity of 0.95 for CT, and a sensitivity of 0.71 and specificity of 0.95 for MRI (Table 5).

**Table 3:** Contingency table analysis of presence of discoid or mixed stenosis for DECT or CT with MRI as standard of reference.

	MRI+	MRI−	Total		MRI+	MRI−	Total
DECT+	45	42	87	CT+	37	27	64
DECT−	63	411	474	CT−	60	414	474
Total	108	453	561	Total	97	441	538
**SE**	**SP**	**PPV**	**NPV**	**SE**	**SP**	**PPV**	**NPV**
0.42 (0.32–0.51)	0.91 (0.88–0.94)	0.52 (0.42–0.62)	0.87 (0.84–0.90)	0.38 (0.28–0.48)	0.94 (0.92–0.96)	0.58 (0.46–0.70)	0.87 (0.84–0.90)

Data are given with 95% confidence intervals.

*SE* sensitivity, *SP* specificity, *PPV* positive predictive value, *NPV* negative predictive value, *MRI* magnetic resonance imaging, *CT* computed tomography, *DECT* dual-energy computed tomography, + discoid or mixed stenosis, − no stenosis or osseous stenosis.

**Table 4:** Contingency table analysis of presence of osseous or mixed stenosis for DECT or MRI with CT as standard of reference.

	CT+	CT−	Total		CT+	CT−	Total
DECT+	72	43	115	MRI+	48	37	85
DECT−	30	397	427	MRI−	54	399	453
Total	102	440	542	Total	102	436	538
**SE**	**SP**	**PPV**	**NPV**	**SE**	**SP**	**PPV**	**NPV**
0.71 (0.62–0.80)	0.90 (0.87–0.93)	0.63 (0.54–0.72)	0.93 (0.91–0.95)	0.47 (0.37–0.57)	0.92 (0.89–0.95)	0.56 (0.45–0.67)	0.88 (0.85–0.91)

Data are given with 95% confidence intervals.

*SE* sensitivity, *SP* specificity, *PPV* positive predictive value, *NPV* negative predictive value, *MRI* magnetic resonance imaging, *CT* computed tomography, *DECT* dual-energy computed tomography, + osseous or mixed stenosis, − no stenosis or discoid stenosis.

**Table 5:** Contingency table analysis of presence of mixed stenosis for DECT, MRI, and CT with both MRI and standard CT as standard of reference.

	Mixed+	Mixed−	Total
DECT+	23	34	57
DECT−	33	435	468
Total	56	469	525
**SE**	**SP**	**PPV**	**NPV**
0.41	0.93	0.40	0.93

	Mixed+	Mixed−	Total
MRI+	40	24	64
MRI−	16	457	473
Total	56	481	537
**SE**	**SP**	**PPV**	**NPV**
0.71	0.95	0.63	0.97

	Mixed+	Mixed−	Total
CT+	22	11	33
CT−	34	472	506
Total	56	483	539
**SE**	**SP**	**PPV**	**NPV**
0.39	0.98	0.67	0.93

Data are given with 95% confidence intervals.

*SE* sensitivity, *SP* specificity, *PPV* positive predictive value, *NPV* negative predictive value, *MRI* magnetic resonance imaging, *CT* computed tomography, *DECT* dual-energy computed tomography, + mixed stenosis, − discoid, osseous, or no stenosis.

Fleiss’ kappa for overall interrater reliability between the readings was 0.321 (p < 0.001) for DECT, 0.254 (p < 0.001) for CT, and 0.281 (p < 0.001) for MRI.

## Discussion

This is the first study investigating the diagnostic accuracy of collagen-sensitive mapping using DECT in the detection of lumbar foraminal stenosis in comparison with MRI and conventional CT. Our results show moderate to good diagnostic accuracy for both DECT (0.71 sensitivity and 0.91 specificity) and conventional CT (0.82 sensitivity and 0.96 specificity) in determining whether a foraminal stenosis is present using MRI and CT as standard of reference. DECT (0.42 sensitivity and 0.91 specificity) and conventional CT (0.38 sensitivity and 0.94 specificity) had low sensitivity but high specificity in detecting discoid stenoses. DECT was found to be more accurate in detecting osseous stenoses compared with MRI (0.71 sensitivity and 0.90 specificity, DECT; 0.47 sensitivity and 0.92 specificity, MRI).

Interrater reliability regarding the degree of foraminal stenosis was good for all three imaging techniques investigated (ICCs: 0.820, MRI; 0.778, DECT; 0.769, CT). At the same time, interrater reliability regarding the type of foraminal stenosis was fair for all three imaging modalities, with lowest agreement for CT (Cohen’s kappa: 0.281, MRI; 0.321, DECT; 0.254, CT). The fact that the analysis regarding the degree of foraminal stenosis yielded the highest interrater agreement for MRI is most likely attributable to the more frequent use of MRI in clinically diagnosing foraminal stenoses. Moreover, the only widely accepted grading system of lumbar foraminal stenosis is available for MRI, which may explain uncertainties in scoring CT and DECT images. Another reason for the lower sensitivities of CT and DECT may be that the grading system by Lee et al. is partly based on the obliteration of perineural fat, which is better depicted by MR imaging^6^. Recently, Haleem et al. developed a novel CT-based classification for foraminal stenosis and show near-perfect agreement with the MRI-based grading system. However, their methodology in evaluating this novel classification is not clear and validation by other authors is still lacking^17^.

Analysis of intermodality agreement in detecting discoid stenoses yielded fair agreement both between MRI and DECT (Cohen’s kappa: 0. 350, p < 0.001) and between MRI and CT (Cohen’s kappa: 0.369, p < 0.001) and in the detection of mixed stenoses fair agreement between MRI and DECT (Cohen’s kappa: 0.336, p < 0.001) and moderate agreement between MRI and CT (Cohen’s kappa: 0.452, p < 0.001). This, in conjunction with the similar diagnostic accuracies, suggests that DECT does not provide additional information for detecting discoid stenoses compared with conventional CT. Even though it is important to bear in mind the lack of an established classification system and that scoring therefore was not standardized, we confirm that standard CT is inferior to MRI in detecting discoid stenoses and show that collagen-sensitive maps derived from DECT do not improve diagnostic accuracy. The rather low intermodality agreement between DECT and standard CT in detecting osseous stenoses may be explained by stenoses that were scored as osseous by standard CT but were found to be mainly discoid in DECT.

To date, DECT has mostly been included in the diagnostic algorithm for patients with gout. Recent studies, however, have shown high sensitivity and specificity and higher interrater agreement of collagen-specific mapping based on DECT in assessing lumbar disc pathologies compared with conventional CT^15^. Furthermore, it was shown that color-coded DECT virtual noncalcium series improved the diagnostic accuracy in detecting cervical disc herniation and spinal nerve root impingement compared with standard CT^18^ and has potential as an imaging biomarker of lumbar intervertebral disc degeneration^19^. Moreover, an added value over conventional CT imaging has been shown in detecting traumatic intervertebral disc injuries, malignant bone marrow infiltration, and posttraumatic bone marrow lesions^9,20–23^. While in a previous study investigating the diagnostic accuracy of collagen-sensitive maps in depicting disc pathologies, foraminal affection was analyzed as well, no foramen-specific results are presented. Thus, while high diagnostic accuracy in determining the presence of disc pathology is shown for collagen-sensitive maps, this study does not allow any conclusions to be drawn regarding their value in depicting foraminal stenosis^14^. Despite the existing clinical applications of DECT, our results show that in detecting discoid lumbar foraminal stenoses, neither CT alone nor CT with collagen-sensitive maps based on DECT are as sensitive or specific as MRI. Thus, whenever these types of stenosis are suspected, MRI remains the standard of reference.

Some limitations need to be discussed. Our analysis is limited by retrospective data collection of a population of patients who did not undergo imaging for symptoms of radiculopathy. Therefore, special transverse images were not available for all foramina, and T2-weighted MR images were not included. Also, correlation of imaging findings and clinical symptoms was not possible. Moreover, some neuroforamina could not be evaluated due to spinal implants and/or disc replacement, especially on MR images. However, as our sample size was still large, we are confident our results are significant, nonetheless. Furthermore, it is important to note that our results are not necessarily transferable to different DECT techniques. As we only included cases of lumbar foraminal stenosis, our results cannot be transferred to the cervical spine.

In conclusion, our results suggest that collagen-sensitive dual-energy reconstruction does not provide additional information to standard CT in evaluating lumbar foraminal stenoses. MRI therefore remains the primary diagnostic tool whenever discoid foraminal stenosis is the suspected cause of lumbar radiculopathy.

## Data Availability

The datasets generated during and/or analysed during the current study are available from the corresponding author on reasonable request.
